# Pauperism, Hospitals

**Published:** 1849-10

**Authors:** W. E. Horner, Samuel Jackson, Alfred Stille, William V. Keating, J. Devereux, James M. Smith, Mark Devine, Joseph Diamond, R. F. Walsh, Robert Ewing

**Affiliations:** Committee; Committee; Committee; Committee; Committee; Committee; Committee; Committee; Committee; Committee


### EDITORIAL DEPARTMENT.

*Pauperism. Hospitals.*—The subject of charitable relief for the indigent sick, is one which it belongs peculiarly to the Physician to investigate and discuss. The practioner of medicine, by his daily professional avocations, necessarily becomes more conversant than other classes of society, (the Clergy, perhaps, excepted), with the sufferings and wants incident to poverty and disease. It is to be expected, therefore, that he will be more competent, than most others, to judge respecting the adequateness and appropriateness of means employed for the relief of those of our fellow beings who are thus doubly afflicted, and, also, of the efficacy of measures designed to prevent the evils of this two-fold calamity. The subject is one which commends itself to the attention and feelings of most members of the medical profession, for, notwithstanding the charge that constant familiarity with suffering begets indifference, we do not hesitate to assert, that by no other class of society, as a class, are the appeals of indigence and illness more deeply felt, and by no other class are they more heartily responded to. The history of almost every successful institution, or scheme for charitable relief, wherever situated, will illustrate the correctness of the foregoing assertion. Few, inded, are the instances in which medical men have not, on such occasions, contributed their share of philanthropic interest, and active service.

We make no farther apology for, or introduction to a few discussive remarks on Pauperism, and Hospitals. What we shall say, (as usual), will be said with the intent only of presenting some ideas for the consideration of our readers. We shall make no attempt to treat of the subject comprehensively or elaborately. For this the medical Journalist seldom has time or space, whatever may be the theme of his lucubrations.

Circumstances, heretofore, have led us to bestow considerable reflection on the subject of pauperism and poor-houses, and, we may add, our opportunities for personal observations in this department of charitable relief have not been inconsiderable. We are satisfied that the entire system as adopted in this country generally, at all events by this State, is radically wrong, founded upon erroneous principles, useless, and even injurious in its results. This opinion is expressed strongly, and, the reader may think, rather rashly. We will briefly state some of the reasons for such an opinion.

We submit, in the first place, that pauperism has no valid, intrinsic claims to countenance and support in our country. The fact of its existence should be considered a libel upon our free institutions, and instead of being protected and provided for by legal statutes, it should not (in our opinion), be recognized by law, or, if any action be had upon it, prohibited, on the same principle that alms-asking is generally made a penal offence.

This position can be sustained in two ways. First, theoretically, it is a legitimate deduction from the nature of our civil and political organization, which is supposed to involve absence of unequal legislation, as well as of hereditary privileges, and aristocratical monopolies; the excess of soil over the numbers for its cultivation, being, at the same time, taken into account. It seems to us susceptible of proof, that if pauperism should, at any time, be developed under our government sufficiently to call for legislative provisions, the fact is evidence of something wrong in the government itself, and that the latter is the place for the radical remedy to be applied. But the limits and scope of this article preclude the discussion of the question in this aspect. Let us then direct our attention to a more practical view, and take into consideration a few facts bearing on the subject.

Of the number of paupers receiving relief in this county, (and, so far as we know, the same is true of other places), by far the greater portion are foreigners. According to the report of the Superintendents of the Poor for the year ending October, 1848, but 153 out of 984, (the whole number relieved at the poor-house), were native Americans. If from the indigenous paupers, we were to deduct those who should have been sent to an hospital rather than to a poor-house, the number would probably be diminished by, at least, one-half. Now we suggest, as a questionable proposition, whether we are bound to maintain *foreign* paupers *as paupers?* It may be assumed that we are under no obligations to do this, inasmuch as we have had no agency in making them such, but they have become so from the unequal influences of other governments. It may be said, with, at least, a show of plausibility, that it is quite enough if we offer them a free government, equal laws, and every facility to derive sustenance from our common mother earth. But a better reason for denying this privilege is, that it is very doubtful, if, in reality, it is charitable, either in the abstract, or in its individual application to the greater portion of the individuals themselves, to provide for their support as paupers. The public are not generally aware of the fact, (which would hardly be credible to one who had not satisfied himself of it by observation), that a considerable proportion of the foreigners who become inmates of our poor-houses, are not paupers by necessity, so much as by inclination. Their associations and aspirations tend to pauperism as the cherished social condition of life. They are not conscious of any sense of degradation, but calculate upon a poor-house, not only as an available resource, but as a desirable attainment, as much as some other individuals look forward to a pecuniary competency as the crowning success of their labors. These persons are paupers by education and profession. Some emigrate with no other intention than to become candidates for our poor laws. Nor is this so strange, when we consider that if they were not paupers at home, their condition was nearly as low in the social scale, and far less comfortable. Compare the situation of our meat-fed, well-housed, warmly-clad, no-worked pauper, with the half-starved, cellar-lodged, semi-naked, over-worked factory operative, in the country of our ancestors—the richest kingdom on the globe, and the fact just stated is not so amazing as it may at first appear to be. We have said that it is doubtful if it be charitable to offer this class of persons the advantage of pauper asylums. They are not wholly responsible, it is true, for the views and feelings they have acquired under other governments; but instead of confirming or encouraging such views and feelings under our government, would it not be a wiser and better policy, and should we not confer on them a more substantial benefit, by endeavoring to teach them to respect themselves as independent, self-relying citizens? Let us offer them a hearty welcome, free institutions, and every encouragement and assistance to render themselves honorable, useful, and happy. If they are afflcted with disease, it is our duty to afford retreats, or hospitals, where every means will be employed for their restoration to health; but let us say to them, we cannot receive you as paupers—pauperism has no place here. It belongs on other shores!

The effect of our present poor-system upon the immigration of foreign paupers, or of those who have no reluctance to become candidates for our poor-houses, is deserving attention. It operates, in fact, as a bounty on the constant importation of that class of persons; and the consequence is, that pauperism is steadily and largely on the increase.

The expenditure required to sustain the present poor laws, is the occasion of incidental evils of no small magnitude, acting as a preventive of, or substitute for, other modes of charitable relief, which are unobjectionable, and would be far more useful. Our hospitals, orphan asylums, etc., cannot be sustained by taxation, because the poor-fund is already so large as to be considered burthensome; and the tax-payer refuses to make voluntary subscriptions for these objects, because he is taxed so heavily for the support of the poor. He considers his conscience discharged of the claims of charitable relief by the requisitions of the law.

But to abolish the present system, it may be said, would interfere with tho claims of a certain portion of those who seek refuge in pauperism. A very small proportion of the inmates of our poor-houses, it will be admitted, are driven there by a hard necessity, which should render them candidates for our warm sympathy and ready protection. But, while this is the case, another portion are made paupers solely by the operation of the present system. There are, in every community, certain individuals who, from degradation, recklessness, and improvidence, sooner or later find their way to the poor-house. For these individuals, true charity consists in efforts to develop and strengthen sentiments of independence, self-reliance, and providence. To place at their command the resources of a poor-house, is directly the reverse of this, and if this resource did not exist, it might, with more success, be urged upon them to achieve a higher destiny than pauperism.

But deducting the number of those to whom these considerations will apply, there will still remain a balance in whose behalf, it will be said, the present laws are required, and with reference to whom they are beneficial in their operation. There are, and must be, some whose necessities are to be provided for—who are, in other words, proper subjects for charitable relief. But the number of these is so small that legal enactments are wholly unnecessary. Abolish poor-laws, and private charity, or that of religious, or of voluntary associations, will be sufficiently ample to reach them all; and the latter will have, in addition, this advantage over public charity, viz: the relief will be more considerately bestowed, and be rendered more complete. It is not one of the least of the evils of the present system, that it tends to repress (as already remarked), individual acts of benevolence. The fact that the law makes provisions for pauperism, shuts out, in many instances, all the reciprocal advantages which would flow from individual acts of charity, as well as all interest in, or knowledge of the subject of charitable relief.

With these remarks on pauperism, we will pass to say a few words respecting *hospitals.* For the medical reader, it is quite needless to amplify on the importance of these institutions of charitable relief. It is obvious that sickness demands the most ample provisions for charitable assistance. Whether disease arise from recklessness, improvidence, or vice, the dictates of humanity are, to administer promptly all practicable measures for comfort and restorement. It would be supererogation to advance any arguments on this point. Yet, hospitals for the relief of the indigent and homeless sick, are as rare in this country as poor-houses are abundant. Except in our larger and older towns, they are almost unknown. The city of Buffalo, for example, with a population of over forty thousand, had no hospital until the establishment of the Hospital of the Sisters of Charity, founded by the pious zeal of the Catholic Bishop of this Diocess, with the co-operation of the religious sisterhood, in honor of whom the institution is named. This hospital finds no lack of inmates, notwithstanding some bigotted prejudices on the score of its denominational character. Now the question arises, what became of the class who are now candidates for the relief it affords, before the institution was established? A large proportion here, as in other places without hospitals, were sent to the poor-house.

Saying nothing, at this time, of the poor-house in this county as a place of resort for the sick poor, (we have said something on this topic heretofore, and have more to say hereafter), the question arises, have the public any right to consign to pauperism those who become subjects for charitable relief on account of sickness alone? Our duty to the sick poor, as it seems to us, is not performed by extending to them the relief afforded by the pauper system, (assuming that this relief were ever so complete), but by doing all in our power to save them from the degradation of being paupers. Although pauperism may not involve guilt, the truth is, it is more disgraceful in public estimation than many species of crime. In so far as the effect upon social position is concerned, the stigma of certain acts of fraud or dishonesty, would, we fear, be elected by many, in preference to the imputation of having been a pauper. Such being the case, the humanity of tendering, as the only resource in sickness coupled with poverty, a poor-house, is more than questionable. It may well be doubted if that be true charity which requires, as the condition of relief, sacrifice of character and self-respect. But there is no alternative in places where poor-houses take the place of hospitals, sickness with poverty, at the same time, abounding beyond the capacity of individual acts of benevolence to provide for it. Hence, another reason why we advocate the abolition of pauperism, and, as a substitute therefor, the same expenditure for public hospitals.

The medical reader may say, why address these remarks to me. This matter does not lie within the power of the medical profession to alter and improve. True, but if the views commend themselves to the approbation of medical readers, they can do much toward effecting the object, by informing, and in that way, directing the opinions of those by whom such changes of improvements are to be effected. The reflecting medical reader will, moreover, perceive, that the subject not only involves the direct benefits of charitable relief, (in which point of view it falls, as already remarked, peculiarly within the province of the physician), but it has important relations to medical science, and medical education; since it is, confessedly, in well-regulated hospitals that scientific observations are best prosecuted, and clinical instruction most successfully conveyed. Let legal provisions for charitable relief assume the direction proposed, leaving the terms *pauper* and *poor-house* to become by-gone expressions of reproach, and, not only our cities, but every county seat, and large village, will have, their sanitary retreats, where the suffering sick may receive appropriate care and skill, and be again restored to society without forfeiture of self-respect, or abatement of the respect of others; and, as incidental philanthropic results, young men preparing for the duties of the medical profession, will have accessible and abundant opportunities for the study of disease at the bed-side, before assuming the responsibilities of its management as practitioners.

But before this can be accomplished, (if it ever can be), much may be done by the organization of hospitals by means of associated effort, irrespective of legal endowment. Let beginnings be made, and opportunities presented for philanthropic persons to witness the good which may in this way be effected. Many will thereby be incited to liberal contributions, or bequests, for an object so purely, and entirely benevolent. We have an illustration of this, in the fact that in cities where hospitals have been for a long time established, they are augmented, and endowed, more and more, by constantly accumulating donations. The palpable evidences of the relief afforded to human suffering, together with the influence of worthy precedents, and a noble spirit of emulation, tend to divert a portion of the superabundant wealth of charitable and generous persons into this channel.

When means cannot be procured for establishing institutions in which medical relief is furnished gratuitously, much charity may be extended by affording a retreat in sickness, with proper medical and other attentions, at a small rate. Upon this plan the Buffalo Hospital of the Sisters of Charity was commenced. The building, fixtures, etc., were procured through the liberality of benevolent persons here, and in other places; the services of the Sisters of Charity were obtained, (of course, without compensation), and a minimum price, for charity patients, was fixed as low as possible to secure a reimbursement for the excess of actual expenses over what other receipts might be calculated upon for the support of the Institution.

This plan has, we perceive, been adopted by an Institution lately established in Philadelphia, connected with which are some of the medical Fraternity of that city, whose names are a sufficient guaranty for the excellency of all that pertains to its medical administration. As the circular of this Institution contains views pertinent to the subject just mentioned, we present it to our readers by way of peroration to this article, omitting some paragraphs which have reference to matters purely local:—

*St. Joseph's Hospital.*—This Institution, chartered by the Legislature of Pennsylvania at its recent session, is now ready for the reception of patients. The site it occupies in the vicinity of the city is admirably adapted, by its beauty and the free ventilation it affords, for the purpose of its selection. On opening the Institution to the public and inviting its support, it may not be regarded as obtrusive or inappropriate, to state the motives and the objects that have governed those by whom it has been founded.

It is most probably unknown to the majority of our fellow citizens, that in the existing population of Philadelphia, rated at 350,000 at least, not more than about one hundred and thirteen beds are provided for sick beneficiaries of the more reputable classes of the poor. From this enumeration are excluded those at the Philadelphia Alms House, and those at the Wills' Hospital, about thirty, devoted to a special service. Upon the principle, therefore, of that common benevolence which inclines many of the benevolent to sympathize in the privations and distresses of others less fortunate than themselves; a place existed for the introduction of a new charity, in addition to the many noble philanthropic institutions already established. It is remarkable that while benevolence has been so active in the final distribution of property by Will, for educational and other meritorious objects, the numerous class of sufferers whom accident or unavoidable causes have prostrated by sickness, has, notwithstanding the injunctions of our Saviour and of the Divine Word, been almost forgotten. "The poor you have always with you," is an enduring fact in the social organization, and not less permanent is the obligation of others attached to it by the precepts of our holy religion. Sickness is unavoidable to the lot of man. No industry or prudence can or will, in every case, guard against this most common of human ills. It is, then, with the view of mitigating the calamities of disease, of accidents and other bodily institutions, in a meritorious portion of our fellow citizens, that the Saint Joseph's Hospital has been instituted.

The benefits to result from a charity of this kind, are strongly illustrated in the case of deserving journeymen, domestics in families, and operatives of various kinds. In addition to the loss of their wages by a suspension of employment in time of sickness, the expenses as connected with disease—as boarding, lodging, nursing, and medicines—are either beyond their means, or are very inadequately supplied. It often happens, that a spell of illness, by the disbursements it produces, sweeps away all the hoarded earnings of an industrious family; and not seldom, the debts incurred in addition, with the long disability for daily exertion, plunge it into an afflicting poverty. The expenses attending a mere attack of disease cannot, on an average, be placed lower than five dollars a week, the final provision for the payment of which must depend upon the duration of the sickness. Hence it happens that an almost irreparable decline in circumstances is sometimes produced. One of the objects of the St. Joseph's Hospital is, by a low charge for the accommodation of such cases, to abridge as much as possible the calamities connected with them. It is believed that this Institution can, for three dollars a week meet all the requisitions of sickness in a more complete manner than can be accomplished in a private house at five.

With the view of carrying out, in the most efficient manner, the objects alluded to, this Hospital has been placed under the charge of a religious Order. The Order chosen is the Sisters of St. Joseph, instituted in Lyons, France, who by their vows make attendance on the sick part of their duty, and who to the desires of their heart to perform it well and faithfully, unite a large amount of experience.

In the present untried state of this Hospital, where a large annual interest is to be paid, it is difficult to foresee what number of gratuitous patients can be received. It is, believed, however, that house rent once free, one beneficiary can be maintained for every five patients at three dollars a week, and if the friends of beneficiaries could contribute something themselves, of course the number would be proportionably increased. * *

Although this Institution has been brought forward under the auspices of one religious denomination, neither its principles nor its charter limit its efforts at doing good to that communion alone, but it is intended, as expressed in the words of the charter, that, "its benefits and advantages shall be extended to the sick, without reference to *creed, country,* or *color.* *

Should the above summary of the character of the St. Joseph's Hospital have the good fortune to obtain the public approbation and sympathy in regard to its objects, it may be scarcely necessary to state, that any contributions in the way of supplies and of subsciption will be gratefully received by its inmates and managers, for it has no other source of existence than the good will of those around it.

Benefactions addressed to the Saint Joseph's Hospital, and left either there or with any of the Managers, will be acknowledged and placed to their proper destination, or should the party intimate a desire to that effect, he will be waited on by some member of the Board of Managers or Physicians.

| | |
|---|---|
| W. E. Horner, M. D., | Samuel Jackson, M. D., |
| Alfred Stille, M. D., | William V. Keating, M. D., |
| J. Devereux, | James M. Smith, |
| Mark Devine, | Joseph Diamond, |
| R. F. Walsh, | Robert Ewing, *Committee.* |

